# Ventilatory support in critically ill hematology patients with respiratory failure

**DOI:** 10.1186/cc11438

**Published:** 2012-07-24

**Authors:** Rosario Molina, Teresa Bernal, Marcio Borges, Rafael Zaragoza, Juan Bonastre, Rosa María Granada, Juan Carlos Rodriguez-Borregán, Karla Núñez, Iratxe Seijas, Ignacio Ayestaran, Guillermo M Albaiceta

**Affiliations:** 1 Servicio de Medicina Intensiva, Hospital Universitario Central de Asturias, Celestino Villamil, 33006 Oviedo, Spain; 2 Servicio de Hematología y Hemoterapia, Hospital Universitario Central de Asturias, Celestino Villamil, 33006 Oviedo, Spain; 3 Servicio de Medicina Intensiva, Hospital Son Llatzer, Ctra Manacor, 07190 Palma de Mallorca, Spain; 4 Servicio de Medicina Intensiva, Hospital Universitario Dr. Peset, Av de Gaspar Aguilar 90, 46017 Valencia, Spain; 5 Servicio de Medicina Intensiva, Hospital Universitario La Fe, Bulevar Sur, 46026 Valencia, Spain; 6 Servicio de Medicina Intensiva, Hospital de Bellvitge, Feixa Larga, 08907 Barcelona, Spain; 7 Servicio de Medicina Intensiva, Hospital Universitario Marqués de Valdecilla, Av de Valdecilla 25, 39008 Santander, Spain; 8 Servicio de Medicina Intensiva, Hospital de la Santa Creu i Sant Pau, UAB, Sant Antoni Maria Claret 167, 08025 Barcelona, Spain; 9 Servicio de Medicina Intensiva, Hospital de Cruces, Plaza de Cruces, 48903 Barakaldo, Bizkaia, Spain; 10 Servicio de Medicina Intensiva, Hospital Universitario Son Espases, Ctra de Valldemossa 79, 07010 Palma de Mallorca, Spain; 11 Departamento de Biología Funcional, Universidad de Oviedo, IUOPA, Julian Clavería, 33006 Oviedo, Spain; 12 CIBER-Enfermedades Respiratorias, Instituto de Salud Carlos III, Spain; 13 Grupo de Trabajo de Enfermedades Infecciosas, Sociedad Española de Medicina Intensiva, Crítica y Unidades Coronarias (SEMICYUC), Spain

## Abstract

**Introduction:**

Hematology patients admitted to the ICU frequently experience respiratory failure and require mechanical ventilation. Noninvasive mechanical ventilation (NIMV) may decrease the risk of intubation, but NIMV failure poses its own risks.

**Methods:**

To establish the impact of ventilatory management and NIMV failure on outcome, data from a prospective, multicenter, observational study were analyzed. All hematology patients admitted to one of the 34 participating ICUs in a 17-month period were followed up. Data on demographics, diagnosis, severity, organ failure, and supportive therapies were recorded. A logistic regression analysis was done to evaluate the risk factors associated with death and NIVM failure.

**Results:**

Of 450 patients, 300 required ventilatory support. A diagnosis of congestive heart failure and the initial use of NIMV significantly improved survival, whereas APACHE II score, allogeneic transplantation, and NIMV failure increased the risk of death. The risk factors associated with NIMV success were age, congestive heart failure, and bacteremia. Patients with NIMV failure experienced a more severe respiratory impairment than did those electively intubated.

**Conclusions:**

NIMV improves the outcome of hematology patients with respiratory insufficiency, but NIMV failure may have the opposite effect. A careful selection of patients with rapidly reversible causes of respiratory failure may increase NIMV success.

## Introduction

Patients admitted to the intensive care unit (ICU) with hematologic malignancies are at high risk of death. Up to 15% of patients with acute leukemia [1] and 20% of those undergoing bone marrow transplantation [2] may require ICU admission. The presence of multiple organ failure in this population has been associated with very high mortality rates [3]. Acute respiratory failure is one of the most prevalent organ failures [4], being the cause of ICU admission in up to 40% [5]. Although mechanical ventilation is the main supportive therapy for those with severe gas-exchange impairment, the need for intubation has been consistently described as one of the most adverse factors in these patients [6,7].

Some reports suggest that the prognosis of these patients has improved in recent years [8,9], although this finding has not been constant among different series [5]. These improvements in the care of hematology patients have led to broadening ICU admission policies [10]. The different changes in the standard of care causing this improvement include the application of noninvasive mechanical ventilation (NIMV) in selected patients, which could avoid the need for intubation and has been shown to decrease mortality [11-13]. Conversely, failure of noninvasive ventilation may lead to a delay in intubation and further impairment in organ failures. This has been found in a randomized trial involving a mixed ICU population [14]. Moreover, observational studies in hematology patients have shown a similar result [6].

Therefore, the type of respiratory support is one of the main prognostic factors in hematology patients admitted to the ICU. To clarify further the role of different ventilatory strategies in the outcome, we conducted an analysis in a large cohort of these patients. Our objective was to establish the risk of death associated with the initial ventilatory approach (either invasive (IMV) or noninvasive) and with the failure of noninvasive ventilation. Afterward, we analyzed the variables related to an increased risk of noninvasive ventilation failure.

## Materials and methods

### Study design

The EMEHU study was performed in 34 ICUs in Spain. All the hematology patients admitted to one of the participating ICUs from June 2007 to September 2008 were prospectively included in a database. Data on demographics, underlying disease, main diagnoses at ICU admission, previous and current treatments, comorbidities, APACHE II severity score, supportive measurements, infections, and the clinical course during the ICU stay (including SOFA scores) were collected. Neutropenia was defined as an absolute neutrophil count below 500/mm^3^. Patients were followed up until hospital discharge or death. The study was approved by each hospital's ethics committee, and informed consent for data collection was obtained from each patient's next of kin.

All the patients included in the database who required some form of positive-pressure ventilation were included in the analysis. The cohort was split into two groups according to their first type of positive-pressure ventilation, either invasive or noninvasive. NIMV failure was defined as the need for intubation after an NIMV trial, irrespective of its duration. The main outcome variable was ICU survival.

### Data analysis

Data are expressed as mean (SD) or number (percentage), as appropriate. Data from patients treated with IMV or NIMV from the onset were compared by using T or χ^2 ^tests for continuous or categoric data, respectively. Univariate comparisons between ICU survivors and nonsurvivors were performed by using the Student *t *or χ^2 ^tests. Variables with differences with a *P *value of 0.1 or less were included in a logistic regression analysis. The goodness-of-fit was evaluated with the Hosmer-Lemeshow test. Odds ratios (ORs) with their 95% confidence interval and *P *values were estimated.

A similar method was used for the comparison between patients with a successful NIMV trial and those with NIMV failure: after univariate comparisons, variables with a *P *of 0.1 or less were included in a logistic regression model. SOFA scores from patients from IMV, NIMV success, and NIMV failure were compared by using an ANOVA. *Post hoc *tests were done when appropriate with the Bonferroni correction. A *P *value equal to 0.05 or less was considered significant. All the statistical calculations were done with SPSS 19 software (IBM, Minneapolis, MN, USA).

## Results

### Impact of ventilation in survival

**Figure 1:**
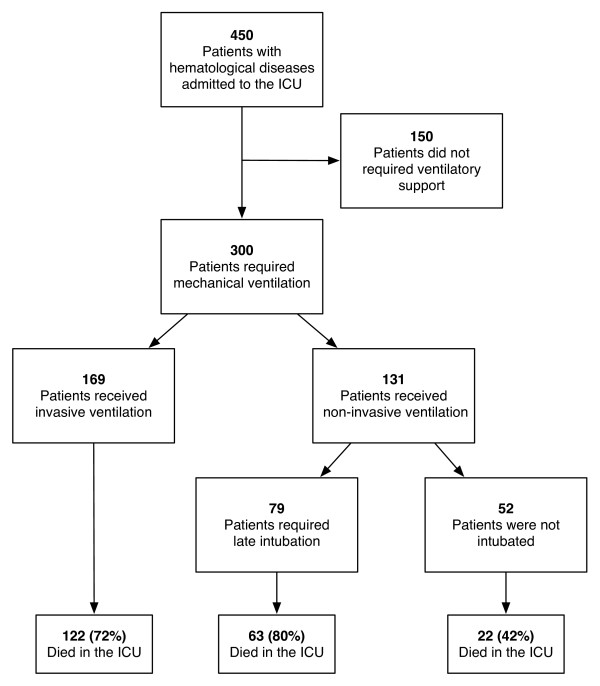
**Study flow chart: patients included in the study, according to the type of respiratory support received**.

Of the 450 hematology patients admitted to the participating ICUs during the study period, 300 (66.7%) required ventilatory support and were included in the analysis. The main demographic and clinical data are shown in Table 1. Overall mortality was 69%. Figure 1 shows the flow chart of the patients included in the study.

**Table 1:** Characteristics of the study sample, comparing intensive care unit survivors and nonsurvivors

	Overall *n *= 300	Survivors *n *= 93	Nonsurvivors *n *= 207	*P *value
Age, years (SD)	53.6 (16.7)	54.9 (14.6)	53.5 (17.5)	0.50

Women (%)	112 (37.5)	41 (36.6)	71 (63.4)	0.12

APACHE II (SD)	24.8 (8.3)	22.18 (7.4)	25.9 (8.3)	0.001

Main diagnosis (%)				0.79

Acute leukemia	127 (44.7)	37 (39.8)	90 (43.4)	

Chronic leukemia	33 (11.6)	9 (9.7)	24 (11.6)	

Hodgkin lymphoma	13 (4.3)	3 (3.2)	10 (4.8)	

Non-Hodgkin lymphoma	76 (26.7)	26 (28)	50 (24.2)	

Multiple myeloma	19 (6.3)	8 (8.6)	11 (5.3)	

Myelodysplastic syndrome	10 (3.3)	2 (2.1)	8 (3.9)	

Other	22 (7.3)	8 (8.6)	14 (6.8)	

HSCT (%)				0.06

No	229 (76.3)	74 (79.6)	155 (74.9)	

Autologous	26 (8.7)	11 (11.8)	15 (7.2)	

Allogeneic	45 (15)	8 (8.6)	37 (17.9)	

Neutropenia (%)	129 (15)	42 (32.6)	87 (67.4)	0.26

Diagnosis on ICU admission (%)				

Shock	81 (27)	18 (19.4)	63 (30.4)	0.05

Infection (other than pneumonia)	100 (33)	35 (37.6)	65 (31.4)	0.29

Pneumonia	112 (37.3)	35 (37.6)	77 (37.2)	0.22

Other causes of respiratory failure	83 (27.6)	21 (22.6)	62 (30)	0.19

Congestive heart failure	20 (6.7)	8 (8.6)	12 (5.8)	0.01

Coma	14 (4.7)	3 (3.2)	11 (5.3)	0.51

Other	52 (17.3)	11 (11.8)	41 (19.8)	0.70

Bacteremia on admission (%)	95 (31.7)	31 (33.3)	64 (30.9)	0.68

Initial ventilatory support (%)				0.17

IMV	169 (56.3)	47 (50.5)	122 (58.9)	

NIMV	131 (43.7)	46 (49.5)	85 (41.1)	

NIMV failure	79 (26.3)	16 (17.2)	63 (30.4)	<0.001

In-hospital days before ICU admission, days (SD)	14.7 (17.3)	15.2 (17.4)	13.4 (16.8)	0.42

Length of ICU stay, days (SD)	11.7 (13.2)	15.7 (16.6)	9.8 (11.0)	<0.001

Values are given as number (%) or mean (SD). HSTC, hematopoietic stem cell transplantation; IMV, invasive mechanical ventilation; NIMV, noninvasive mechanical ventilation.

First, we compared data from patients submitted to IMV as the first option with those from patients who were managed initially with NIMV. Patients in the IMV group showed an increased severity (APACHE II score, 26.1 (8.4) versus 23.1 (7.8), *P *= 0.004), with no differences for age (54.3 (17) versus 53.4 (16.5) years; *P *= 0.91) or sex (39.9% versus 34.4% females; *P *= 0.33). Both groups showed a similar distribution of hematologic diagnoses, rate of neutropenia, and type of hematopoietic stem cell transplantation (HSCT, data not shown). At ICU admission, patients assigned to IMV had higher rates of shock (33.1% versus 19.1%; *P *= 0.007) and coma (7.7% versus 0.8%; *P *= 0.004) and a lower incidence of congestive heart failure (3% versus 10%; *P *= 0.014). These results suggest that the most severely ill patients were electively intubated and subject to IMV.

We then performed univariate comparisons between ICU survivors and nonsurvivors and found that survivors have lower APACHE II scores, lower incidence of shock, and a higher incidence of congestive heart failure on admission (Table 1). As expected, because of the previous finding of increased severity in electively intubated patients, significant differences were present between survivors and nonsurvivors in the type of respiratory support, with lower mortality rates in patients receiving NIMV (42.3%) than in those that were intubated from the onset (72.2%) or after NIMV failure (79.7%). Of the 131 patients managed initially with NIMV, 79 (60.3%) required intubation and invasive ventilation. No differences were found in the day of onset of IMV (0.9 ± 1.5 versus 1.2 ± 1.6 days for survivors and nonsurvivors, respectively; *P *= 0.33) or NIMV (0.4 ± 0.7 versus 0.7 ± 1.2 days for survivors and nonsurvivors, respectively; *P *= 0.14).

All the variables with a *P *value <0.1 were entered into a logistic regression analysis. Goodness-of-fit was adequate, with a *P *= 0.20 in the Hosmer-Lemeshow test. The results of this analysis are shown in Table 2. We found that APACHE II scores, allogeneic stem cell transplantation, and NIVM failure were independent risk factors for increased ICU mortality in this population. However, a diagnosis of congestive heart failure at ICU admission and the initial use of NIMV were factors that reduce the risk of NIMV failure. Adding an interaction between initial ventilatory strategy and NIMV failure did not change the results (data not shown).

**Table 2:** Risk factors for death in the ICU, according to logistic regression analysis

	OR	95% confidence interval	*P*
APACHE-II	1.06	1.02-1.10	0.002

Congestive heart failure on admission	0.26	0.08-0.85	0.026

Shock on admission	1.69	0.86-3.33	0.131

NIMV as first ventilatory approach	0.32	0.15-0.67	0.003

NIMV failure	5.74	2.40-13.73	<0.001

Allogeneic HSCT	6.78	1.78-25.85	0.005

HSCT, hematopoietic stem cell transplantation; OR, odds ratio; NIMV, noninvasive mechanical ventilation.

Overall, our data demonstrate the critical role of the type of ventilatory support in the prognosis of hematology patients.

### Risk factors for NIVM failure

The previous logistic regression analysis demonstrates that NIMV is associated with an improved outcome of hematology patients requiring ventilatory support, but the failure of NIVM increases significantly the risk of death in this population (with an OR of 5.74), even more than elective invasive ventilation (which results in an OR of 3.13). Then we studied the differences between patients with failure of NIMV and those who do not require intubation. The duration of NIMV was not different between groups (3.7 (4.5) and 3.3 (7.2) days in NIMV success and failure groups, respectively; *P *= 0.7), nor in-hospital stay before ICU admission (13.9 [16.2] and 15.7 [20.2] days; *P *= 0.59). ICU stay was shorter in patients with NIMV success (5.6 (4.8) versus 14 (14.2) days; *P *< 0.001). Table 3 shows the differences in univariate comparisons and the results of the logistic regression. The NIMV-failure group patients were younger and had a lower incidence of congestive heart failure and bacteremia on admission. A trend existed to a significant difference in the distribution of hematologic diagnosis, and acute leukemia and myeloma were less common in the NIMV failure group. In the multivariate analysis, age, diagnosis of congestive heart failure, or bacteremia on admission were risk factors independently associated with a decreased risk of NIMV failure. Again, the goodness-of-fit of the model was adequate (Hosmer-Lemeshow; *P *= 0.31).

**Table 3:** Failure of noninvasive mechanical ventilation

	Univariate comparison			Logistic regression		
	**NIV success** ***n *= 52**	**NIV failure** ***n *= 79**	***P *value** **(univariate)**	**OR**	**95% confidence interval**	***P *value**

Age, years (SD)	58.1 (12.1)	50.4 (18.1)	0.004	0.958	0.932-0.985	0.002

Women (%)	22 (42)	23 (29)	0.13			

APACHE II (SD)	22.3 (6.3)	23.7 (8.5)	0.37			

Main diagnosis (%)			0.07			

Acute leukemia	25 (48)	28 (35.4)		0.606	0.133-2.753	0.52

Chronic leukemia	4 (7.6)	14 (17.7)		3.482	0.531-22.834	0.19

Hodgkin lymphoma	2 (3.8)	6 (7.6)		2.495	0.241-25.835	0.44

Non-Hodgkin lymphoma	10 (19.2)	22 (27.8)		1.451	0.291-7.237	0.65

Multiple myeloma	6 (11.5)	2 (2.5)		0.245	0.028-2.170	0.21

Myelodysplastic syndrome	1 (1.9)	0 (0)				

Other	0 (0)	1 (1.3)				

HSCT (%)			0.60			

No	39 (75)	62 (78.5)				

Autologous	8 (15.4)	13 (16.4)				

Allogeneic	5 (9.6)	4 (5.1)				

Neutropenia (%)	24 (42)	33 (58)	0.54			

Diagnosis on ICU admission (%)						

Shock	12 (23)	13 (16.5)	0.37			

Infection (other than pneumonia)	33 (63)	56 (70.9)	0.44			

Pneumonia	20 (50)	38 (63.3)	0.28			

Other causes of respiratory failure	12 (30.7)	26 (22.8)	0.22			

Congestive heart failure	10 (19.2)	4 (5.1)	0.02	0.162	0.034-0.780	0.02

Coma	1 (1.9)	0 (0)				

Other	3 (5.8)	8 (10.1)	0.38			

Bacteremia on admission (%)	22 (42.3)	19 (24.1)	0.03	0.422	0.179-0.997	0.05

Univariate comparisons between patients with NIMV success and NIMV failure, and results of the logistic regression analysis. Values are given as number (%) or mean (SD).

**Figure 2:**
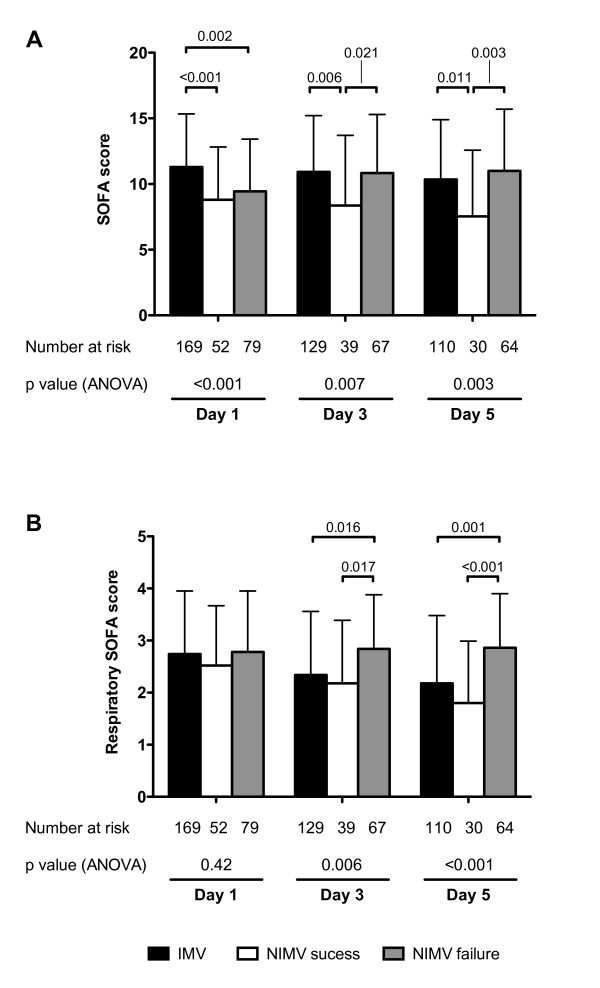
**Organ failure according to ventilatory strategy**. Time course of organ failures, assessed with the SOFA score, in patients treated with elective invasive mechanical ventilation (IMV) and those treated with noninvasive mechanical ventilation (NIMV), divided into those wih success and those with NIMV failure.

To test the hypothesis that NIMV failure could result in additional systemic and respiratory derangement, we compared the SOFA scores in the first 5 days after ICU admission. Patients who were intubated as the first option had higher SOFA scores at day 1. In case of NIVM success, SOFA scores were lower at all time points. However, NIVM failure was associated with scores similar to the IMV group (Figure 2A). When only the respiratory item in the SOFA scale was compared, we found that, in the absence of differences at day 1, the NIVM-failure group values were significantly higher at later times (Figure 2B). No significant differences were found in other items of the score.

## Discussion

The results reported here demonstrate the critical role of the type of respiratory support in hematology patients. In particular, the use of NIMV may have a dual effect: its application results in a significant decrease in mortality, but the failure after a NIMV trial increases the risk of death. Additionally, we identified other factors related to a poor outcome (severity, cause of respiratory derangement) and NIMV failure. In contrast, a diagnosis of congestive heart failure was related to improved outcomes.

### Prognosis of hematology patients in the ICU

Hematologyl patients differ from other cancer patients admitted to the ICU in their more-serious condition and higher mortality rates, which may be as high as 40% to 50% [15]. Some of the prognostic factors found in our study are consistent with previously published results. Severity scores have been almost universally linked to ICU outcome [16]. As the focus of our study was on respiratory failure and its treatment in this population, all the other significant variables were related to the cause of lung injury or the type of respiratory support. The patient selection may also explain the fact that other variables commonly related to outcome, such as days before ICU admission [17], were not significant in our model.

### Impact of respiratory failure and its treatment

Increasing evidence suggests that the severity of organ failure and persistence in time are of paramount importance for survival [11,18,19]. Specifically, the presence of respiratory failure aggravates the prognosis and the course of the disease in these patients, with an associated mortality rate of about 50% [4]. This rate may increase up to 75% to 90% when invasive mechanical ventilation is needed [20]. Noninvasive mechanical ventilation has emerged as an alternative with which to avoid intubation. Along this line, several studies published in the last decade attribute to NIMV a protective effect, avoiding intubation in up to half the cases, thus improving outcome [11,21]. Therefore, NIMV has become the preferable initial method of ventilation in this population. Our results show that use of NIMV is related to a decreased overall mortality in hematology patients.

However, this form of ventilation may have some drawbacks. The risk of NIVM failure in hematology patients is high, with rates ranging from 50% to 70% [13,19], in accord with our own result (60%). Previous reports in a mixed ICU population showed an increase in the risk of death after NIMV failure [22]. Similarly, in a prospective, randomized study on the use of NIVM to treat postextubation respiratory insufficiency, failure of NIVM increased the risk of death [14]. Regarding our study population, the logistic regression analysis shows that NIMV failure is an independent predictor of death, with an OR >5. A similar result was found in single-center observational studies [6,17], and results from a large database of hematology patients showed a higher, although nonsignificant, in-hospital mortality in those intubated after an NIVM trial [12]. It has been hypothesized that NIMV failure may delay the onset of optimal respiratory support in these patients. The data that show worse scores in the SOFA item measuring gas exchange support this point, although the lack of standardized criteria for intubation, inherent in the observational nature of the study, precludes any firm conclusion.

Our results highlight the relevance of NIMV failure as an emerging concern when treating hematology patients with respiratory insufficiency, especially because of the increasing use of NIMV in hematology patients [7] and the high failure rates reported.

### Risk factors for NIMV failure

Several different factors lead to NIVM failure. An increased severity of the disease, measured by using scores such as SAPS II or by the number of organ failures, has been associated with increased intubation rates [18,19]. However, we found no differences in the APACHE II in our sample. The cause of the respiratory failure also plays a key role in predicting the success of NIMV. NIVM has been shown to be highly effective in cardiogenic pulmonary edema [23], consistent with previous studies [6] and our results. Conversely, its use in cases of pneumonia, acute lung injury, or those without an identified cause of respiratory failure is more controversial [24]. The presence of acute lung injury as a risk factor of NIVM failure in hematology patients was recently confirmed in a large sample. However, no data regarding congestive heart failure in this cohort were reported [12]. Finally, bacteremia on admission was another factor related to NIMV success [21]. It may be argued that prompt and specific antibiotic treatment, guided by microbiologic results, is strongly related to an improved outcome, as shown by others [25].

Taken together, these results suggest that diseases that may have a fast response to therapy, such as diuretics and inotropes for cardiogenic pulmonary edema or directed antibiotic therapy for a documented infection, may benefit from NIMV. However, in other causes of lung injury, NIMV may not support the ventilatory function for a prolonged time, thus increasing the risk of failure and intubation.

## Conclusions

All of these results highlight the benefits and risks of NIMV in critically ill hematology patients. Based on previously published data and our own results, it seems critical to select candidates carefully for NIMV among those with rapidly reversible causes of respiratory failure (that is, congestive heart failure). In other cases, the delay in optimal respiratory support with IMV may increase the risk of death.

## Key messages

• Noninvasive mechanical ventilation improves survival in hematology patients with acute respiratory failure.

• However, failure of noninvasive ventilation increases mortality, even more than does elective invasive ventilation.

• Success of noninvasive ventilation is higher in conditions with specific therapy, such as congestive heart failure or documented bacteremia.

## Abbreviations

APACHE II: Acute Physiology and Chronic Health Evaluation: Version II; HSCT: hematopoietic stem cell transplantation; ICU: intensive care unit; IMV: invasive mechanical ventilation; NIMV: noninvasive mechanical ventilation; SAPS II: Simplified Acute Physiology Score: Version II; SOFA: Sequential Organ-failure Assessment.

## Competing interests

The authors declare that they have no competing interests.

## Authors' contributions

RZ and MB designed the study. RM, TB, and GMA planned and performed the specific analysis of the data. TB, GMA, MB, RZ, JB, RMG, JCRB, KN, IS, and IA collected the data and discussed the results. TB, RM, and GMA wrote the manuscript. All authors read and approved the final manuscript.
